# Ophiobolin A kills human glioblastoma cells by inducing endoplasmic reticulum stress via disruption of thiol proteostasis

**DOI:** 10.18632/oncotarget.22537

**Published:** 2017-11-20

**Authors:** In Young Kim, MiRi Kwon, Min-Koo Choi, Dongjoo Lee, Dong Min Lee, Min Ji Seo, Kyeong Sook Choi

**Affiliations:** ^1^ Department of Biochemistry, Ajou University School of Medicine, Suwon, Korea; ^2^ BK21 Plus Program, Department of Biomedical Sciences, Ajou University School of Medicine, Suwon, Korea; ^3^ College of Pharmacy, Dankook University, Cheonan, Korea; ^4^ College of Pharmacy, Ajou University, Suwon, Korea

**Keywords:** ophiobolin A, endoplasmic reticulum stress, proteostasis, thiol, paraptosis-like cell death

## Abstract

Ophiobolin A (OP-A), a fungal sesterterpene from *Bipolaris oryzae*, was recently shown to have anti-glioma activity. We show here that OP-A induces paraptosis-like cell death accompanied by dilation of the endoplasmic reticulum (ER) in glioma cells, and that CHOP-mediated ER stress plays a critical role in this process. OP-A-induced ER-derived dilation and cell death were found to be independent of reactive oxygen species, but were effectively blocked by various thiol antioxidants. We observed that OP-A can react with cysteinyl thiols to form Michael adducts, suggesting that the ability of OP-A to covalently modify free sulfhydryl groups on proteins may cause protein misfolding and the accumulation of misfolded proteins, leading to paraptosis-like cell death. Taken together, these results indicate that the disruption of thiol proteostasis may critically contribute to the anti-glioma activity of OP-A.

## INTRODUCTION

Glioblastoma multiforme (GBM), also known as grade IV astrocytoma, is the most common primary malignant brain tumor [1]. Complete resection remains virtually impossible due to the tendency of GBM cells to invade into the brain parenchyma [1, 2]. Despite the use of aggressive surgical resection, intensified radiation therapy, and concomitant chemotherapy with temozolomide, GBM remains a fatal disease with an average survival time of 14.6 months following diagnosis [2]. GBM cells resist apoptosis, largely explaining the low effectiveness of the classical chemotherapeutic approaches that are based on the induction of apoptosis in cancer cells [1, 2]. Therefore, therapeutic strategies to induce non-apoptotic cell death may offer an innovative opportunity to combat GBM. Recently, a new type of non-apoptotic cell death, termed paraptosis, was reported to be induced by several natural products, including curcumin [3, 4], celastrol [5], and paclitaxel [6], and to show anti-cancer activities [7]. Therefore, the identification of agents that induce paraptosis or paraptosis-like cell death, and efforts to understand their underlying mechanisms, may support the development of an alternative therapeutic strategy for overcoming innate and acquired resistance of cancer cells to the current proapoptotic anticancer therapies. Paraptosis is characterized by a process of vacuolation that begins with physical enlargement of the endoplasmic reticulum (ER) and/or mitochondria [7–9]. Paraptosis does not involve the characteristic apoptotic features of pyknosis, DNA fragmentation, or caspase activation; instead, it requires new protein synthesis [8]. Although the molecular bases of paraptosis or paraptosis-like cell death are still not clearly understood, various mechanisms have been proposed, including imbalances in the homeostasis of ions (e.g., Ca^2+^ and K^+^) [4, 5, 10], generation of ROS [3, 11], and perturbation of cellular proteostasis due to proteasomal inhibition [3, 12] and/or disruption of sulfhydryl homeostasis [7, 13, 14].

Recent evidence indicates that Ophiobolin A (OP-A), a sesterterpenoid produced by pathogenic fungi of the genus *Bipolaris* [15], can be used as an anti-cancer agent [16–20]. OP-A may act selectively on cancer stem cells by inhibiting K-ras4B activity through the inactivation of calmodulin [20]. OP-A inhibits tumor growth in both apoptosis-sensitive and apoptosis-resistant cancer cells, as well as in cancer cells that display various multidrug resistance phenotypes [16]. OP-A is considered a promising candidate for treating GBM [10, 21]: in an orthotopic mouse GBM model, it increased survival, decreased tumor growth, and showed an ability to cross the blood-brain-barrier (BBB) [21]. OP-A was also shown to decrease BKCa channel activity and trigger paraptosis-like cell death in human GBM cells [10], although it is not yet known whether BKCa channel inhibition is functionally related to the observed cell death. For the evaluation of OP-A as a potential anti-glioma agent, the underlying mechanisms through which OP-A kills glioma cells need further extensive investigation. We report here for the first time that OP-A commonly induces ER stress in glioma cells, and that CHOP upregulation plays a critical role in OP-A-induced paraptosis-like cell death. Additionally, we provide evidence that the ability of OP-A to covalently modify free sulfhydryl groups on proteins critically contributes to protein misfolding and the accumulation of misfolded proteins within the ER, leading to ER stress, ER dilation, and paraptosis-like cell death in various cancer cell lines. Collectively, our results show that OP-A treatment may provide an effective therapeutic strategy against cancer cells by disrupting thiol proteostasis.

## RESULTS

### OP-A induces paraptosis-like cell death in glioma cells via dilation of the ER

**Figure 1:**
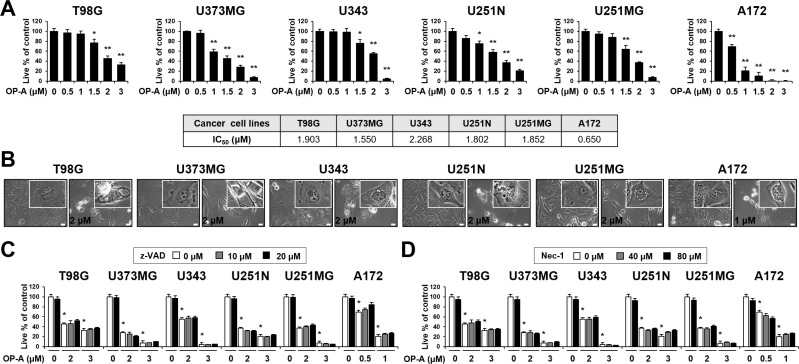
Neither apoptosis nor necroptosis is involved in OP-A-induced cell death in various glioma cells (**A**, **B**) Cells were treated with the indicated concentrations of OP-A for 24 h. (A) Cellular viability was assessed using calcein-AM and EthD-1. Data represent the means ± SD (*n* = 7). One-way ANOVA and Bonferroni’s *post hoc* test. ^*^P < 0.01, ^**^P < 0.001 vs. untreated control. IC_50_s were calculated using GraphPad Prism. (B) Phase-contrast microscopy. Bar 20 μm. (**C**, **D**) Cells were pretreated with z-VAD-fmk (C) or necrostatin-1 (D) for 30 min and further treated with the indicated concentrations of OP-A for 24 h. Cellular viability was assessed using calcein-AM and EthD-1. Data represent the means ± SD (*n* = 7). One-way ANOVA and Bonferroni’s *post hoc* test. ^*^P < 0.001 vs. untreated control.

**Figure 2:**
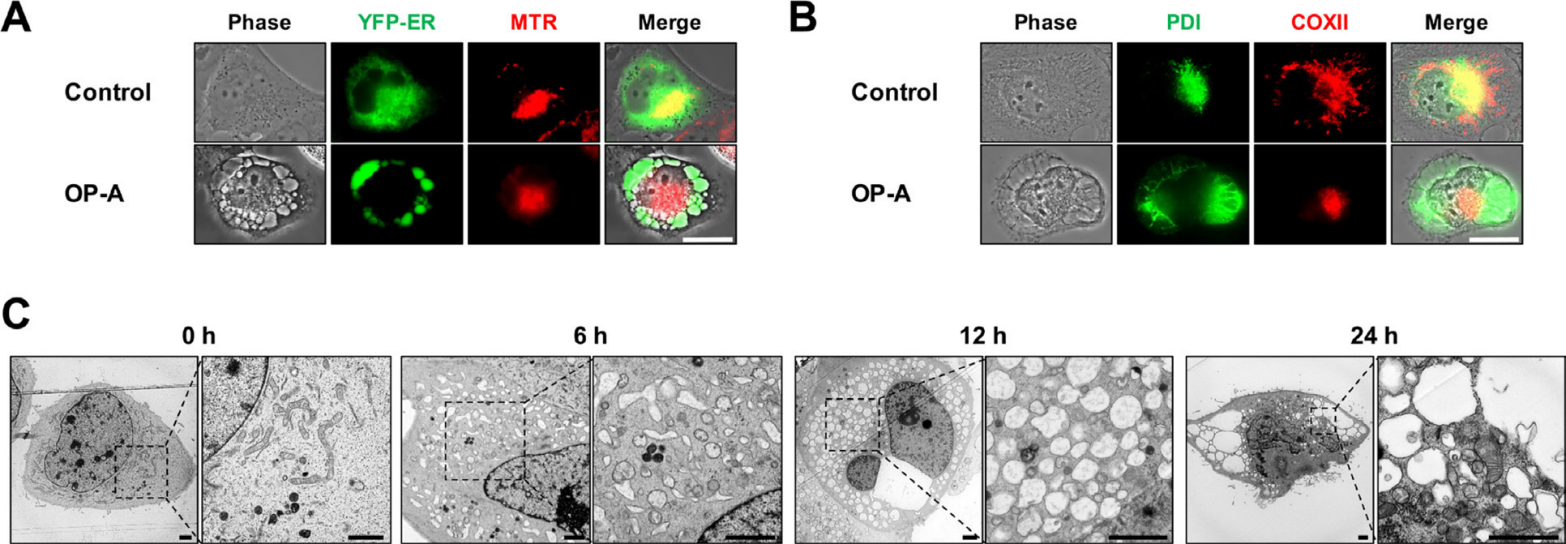
OP-A induces paraptosis-like cell death in various glioma cells (**A**, **B**) Cells were untreated or treated with 2 μM OP-A for 12 h. (A) YFP-ER Cells were stained with Mito-tracker red (MTR) and observed under the phase-contrast and fluorescence microscope. Bar 20 μm. (B) Cells were subjected for immunocytochemistry of COX II and PDI. Bar 20 μm. (**C**) Transmission electron microscopy of T98G cells treated with 2 μM OP-A. Bar 20 μm.

To investigate the mechanism underlying OP-A-induced glioma cell death, we first examined the effect of OP-A on the viability of various glioma cell lines. OP-A treatment dose-dependently reduced the viability of T98G, U373MG, U343, U251N, U251MG, and A172 cells (Figure 1A). Although slight between-line differences in OP-A sensitivity were observed with A172 cells demonstrating the highest sensitivity, the OP-A-induced cell death in these glioma cells was commonly notably accompanied by a marked vacuolation (Figure 1B). When we tested the possible involvement of apoptosis in this process, pretreatment with z-VAD-fmk (a pan-caspase inhibitor) had no effect on OP-A-induced cell death (Figure 1C) or vacuolation (Supplementary Figure 1). Neither caspase-3 nor PARP (a substrate of caspase-3) was cleaved in T98G and U373MG cells treated with OP-A: in contrast, they were cleaved in T98G cells treated with TRAIL (a positive control for apoptosis), and z-VAD-fmk pretreatment effectively blocked TRAIL-induced cell death (Supplementary Figure 2). OP-A-induced vacuolation (Supplementary Figure 1) and cell death (Figure 1D) were also unaffected by pretreatment with necrostatin-1, a specific inhibitor of necroptosis. These results suggest that OP-A-induced cell death in these cells is not associated with apoptosis or necroptosis. To identify the origins of the OP-A-induced vacuolation, we examined the morphologies of the endoplasmic reticulum (ER) and mitochondria using YFP-ER cells (a T98G subline that expresses fluorescence specifically in the ER) and Mito-Tracker Red, respectively. The ER and mitochondria showed reticular and filamentous/elongated morphologies, respectively, in untreated YFP-ER cells; in contrast, OP-A-treated YFP-ER cells for 12 h exhibited green fluorescence (corresponding to the ER) within vacuoles and aggregation of mitochondria adjacent to nuclei (Figure 2A). Immunocytochemical analyses of PDI (an ER resident protein) and COXII (a mitochondrial protein) showed that PDI was mainly expressed at the periphery of the extensively dilated vacuoles in the cytosol, whereas COXII was expressed focally beside the nuclei in T98G cells treated with OP-A for 12 h (Figure 2B). Electron microscopy showed that ER cisternae were distended and mitochondria were shortened in T98G cells treated with 2 µM OP-A for 6 h (Figure 2C). At 12 h, further expansion and fusion of swollen ER were observed, along with a dramatic dilation of the perinuclear space. At time points beyond 12 h, the fusion of the dilated ER progressed further until most of the cellular space was occupied by expanded ER-derived vacuoles. Collectively, these results suggest that OP-A kills glioma cells by inducing a paraptosis-like cell death, in which the cellular vacuolation is mainly derived from the ER.

### OP-A induces ER stress in glioma cells and CHOP plays a critical role in OP-A-induced paraptosis-like cell death of these cells

**Figure 3:**
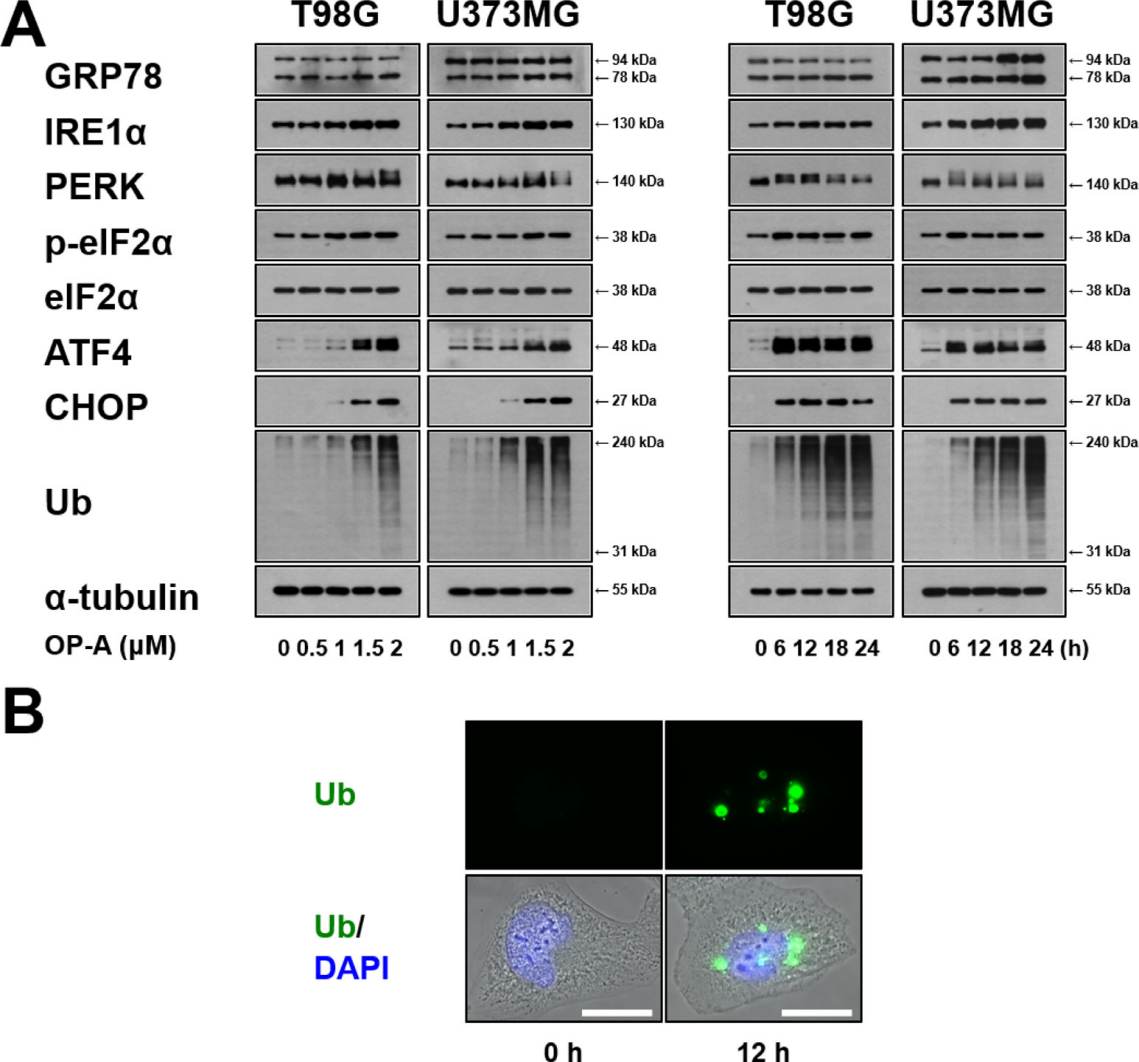
OP-A induces ER stress in glioma cells (**A**) T98G or U373 MG cells were treated with the indicated concentrations of OP-A for 12 h or 2 µM OP-A for the indicated time points and Western blotting of the indicated proteins was performed. α-tubulin was used as a loading control in Western blots. (**B**) T98G cells treated with 2 µM OP-A for the 12 h were fixed, immunostained using anti-ubiquitin antibody (green), and subjected to immunocytochemistry.

**Figure 4:**
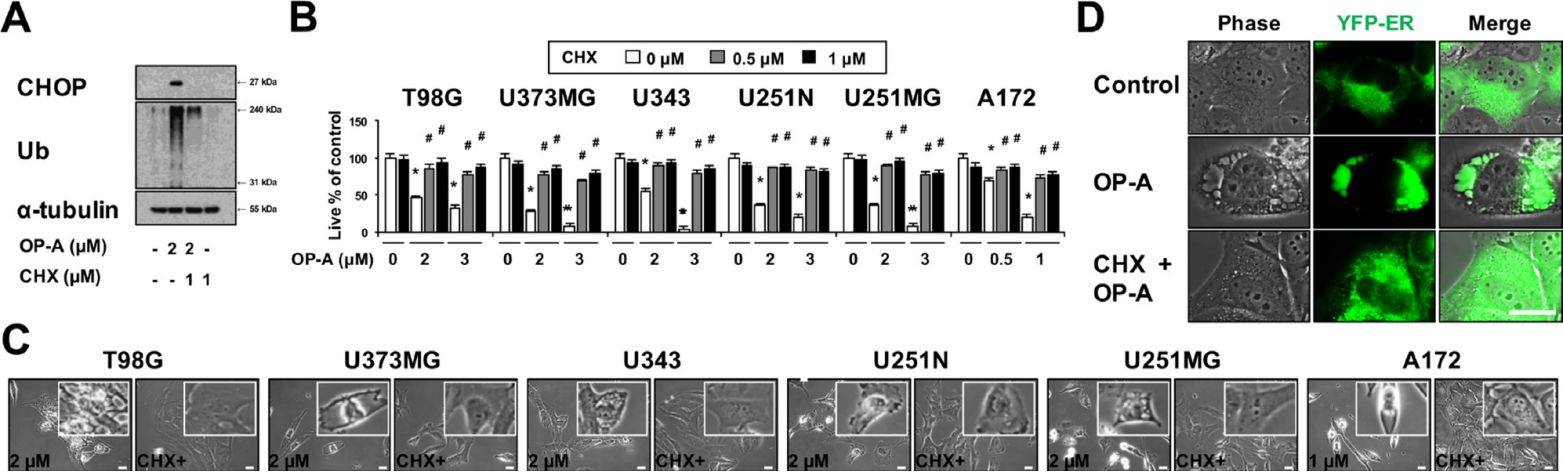
OP-A-induced vacuolation and cell death in various glioma cells are inhibited by CHX pretreatment (**A**) T98G cells were untreated or pretreated with 1 µM CHX and further treated with 2 µM OP-A for 12 h. Western blotting of ubiquitin, CHOP, and α-tubulin was performed. (**B**) Cells were pretreated with the indicated concentrations of CHX and further treated with the indicated concentrations of OP-A for 24 h. Cellular viability was assessed using calcein-AM and EthD-1. Data represent the means ± SD (*n* = 7). Statistical significance was determined using one-way ANOVA followed by Bonferroni’s *post hoc* tests. ^*^P < 0.001 vs. untreated control, ^#^*P* < 0.05 vs. OP-A treatment. (**C**) Cells pretreated with 1 µM CHX and further treated with the indicated concentrations of OP-A for 24 h were observed under the phase-contrast microscope. Bar, 20 μm. (**D**) YFP-ER cells were untreated or pre-treated with 2 μM CHX and further treated with 2 μM OP-A for 12 h. Cells were observed under the phase-contrast and fluorescence microscopy. Bar, 20 µm.

ER dilation was previously shown to be a characteristic response to ER stress that arises due to the accumulation of misfolded proteins in the ER lumen [3, 7, 22]. Therefore, we examined whether OP-A affects the expression of ER stress-related proteins. We found that OP-A treatment of T98G and U373MG cells for 12 h dose-dependently upregulated the protein levels of GRP78, IRE1α, ATF4, and CHOP and increased the phosphorylation levels of PERK and eIF2α (Figure 3A). In addition, treatment with 2 µM OP-A progressively increased the protein levels of GRP78 and IRE1α (Figure 3A). The phosphorylation levels of PERK and eIF2α and the protein levels of ATF4 all increased to a peak at 6 h of OP-A treatment and decreased thereafter; in contrast, the protein level of CHOP was upregulated beginning at 6 h of OP-A treatment and relatively sustained thereafter. Additionally, we found that OP-A treatment triggered the dose- and time-dependent accumulation of poly-ubiquitinated proteins (Figure 3A). Immunocytochemistry also revealed the presence of poly-ubiquitinated protein aggregates in OP-A-treated T98G cells (Figure 3B), indicating that OP-A may disrupt proteostasis. Since paraptosis or paraptosis-like cell death is known to require *de novo* protein synthesis [8, 26], we tested the effect of the protein synthesis blocker, cycloheximide (CHX), on OP-A-induced cellular responses. We found that CHX pretreatment almost completely inhibited the OP-A-induced accumulations of CHOP and poly-ubiquitinated proteins in T98G cells (Figure 4A). In addition, CHX effectively blocked OP-A-induced vacuolation and cell death in all of the tested glioma cell lines (Figure 4B and 4C). The ability of CHX to block OP-A-induced ER-derived vacuolation was also confirmed in YFP-ER cells (Figure 4D). These results suggest that CHX, which arrests *de novo* protein synthesis, reduces the burden on the homeostatic protein-folding mechanisms and significantly delays the OP-A-induced cell death response. Taken together, these results suggest that the treatment of glioma cells with OP-A triggers misfolded protein accumulation, ER stress, and ER dilation, finally resulting in paraptosis-like cell death.

**Figure 5:**
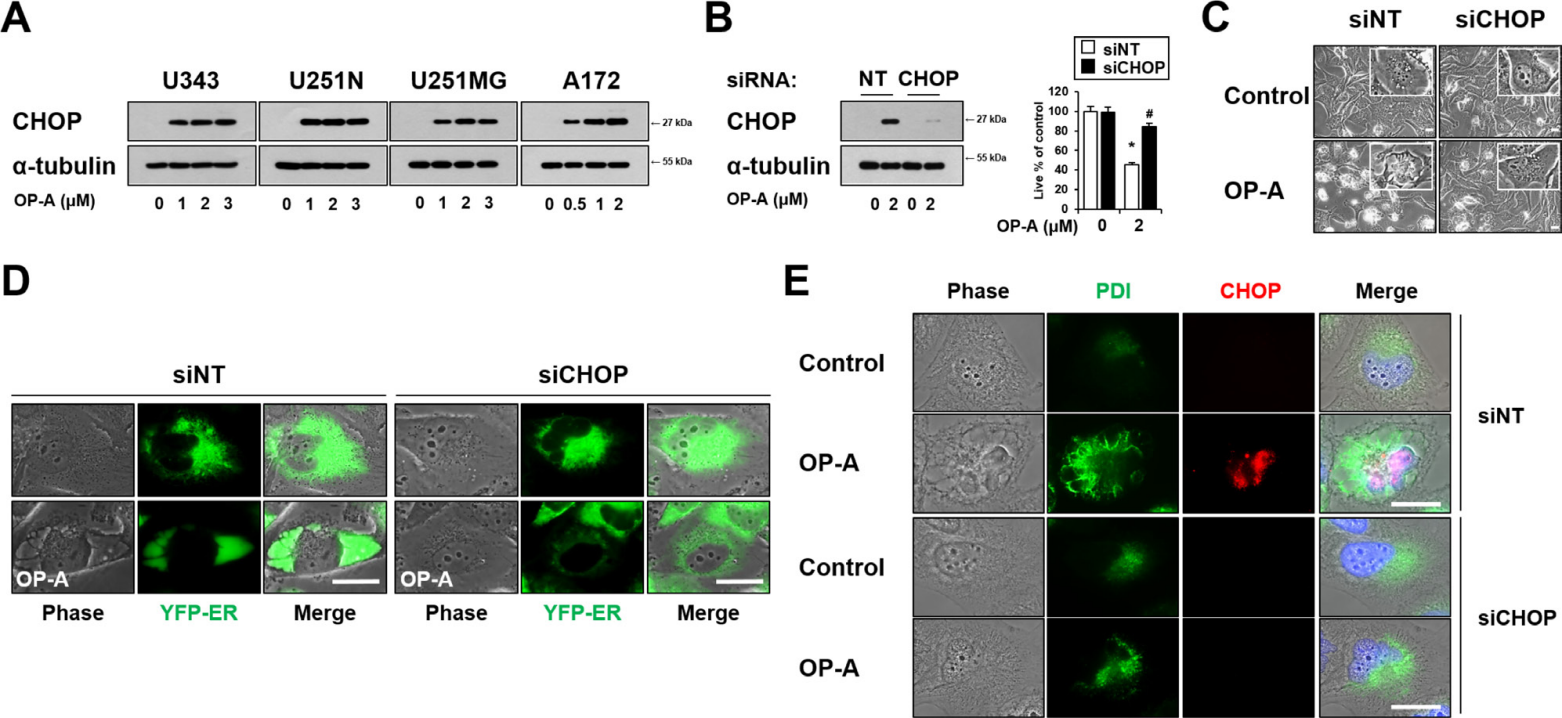
CHOP upregulation plays a critical role in OP-A-induced paraptosis-like cell death (**A**) U343, U251N, U251MG and A172 cells were treated with indicated concentration of OP-A for 12 h and Western blotting of CHOP and α-tubulin was performed. (**B**–**E**) T98G cells transfected with the non-targeting siRNA (siNT) or CHOP siRNA were further treated with 2 μM OP-A for 16 h. (B) CHOP knockdown was confirmed by western blotting (*left*). Cellular viability was assessed using calcein-AM and EthD-1 (*right*). Data represent the means ± SD (*n* = 7). One-way ANOVA and Bonferroni’s *post hoc* test. ^*^P < 0.001 vs. untreated control, ^#^*P* < 0.05 vs. OP-A treatment. (C) Treated cells were observed under the phase-contrast microscope. Bar, 20 µm. (D) YFP-ER cells were transfected with siNT or CHOP siRNA and further treated with 2 μM OP-A for 16 h. Phase-contrast and fluorescence microscopy was performed. (E) Treated cells were subjected to immunocytochemistry of PDI and CHOP. Representative pictures of cells are shown. Bar 20 μm.

As CHOP is involved in making the cell death decision associated with ER stress [23], and OP-A treatment commonly upregulated CHOP protein levels in the tested glioma cells (Figures 3A and 5A), we next tested whether CHOP is critically involved in OP-A-induced paraptosis-like cell death. Indeed, we found that siRNA-mediated CHOP knockdown effectively inhibited OP-A-induced cell death (Figure 5B) and vacuolation (Figure 5C).This CHOP knockdown-mediated inhibition of OP-A-induced ER-derived vacuolation was confirmed by the fluorescence microscopy in YFP-ER cells (Figure 5D), and by immunocytochemical analysis of PDI and CHOP (Figure 5E). Similar results were obtained in experiments performed using CHOP shRNA (Supplementary Figure 3). Collectively, our results indicate that CHOP upregulation plays a crucial role in the OP-A-induced paraptosis-like cell death of glioma cells.

### OP-A-induced paraptosis-like cell death is blocked by various thiol-antioxidants, but not by non-thiol ROS scavengers

**Figure 6:**
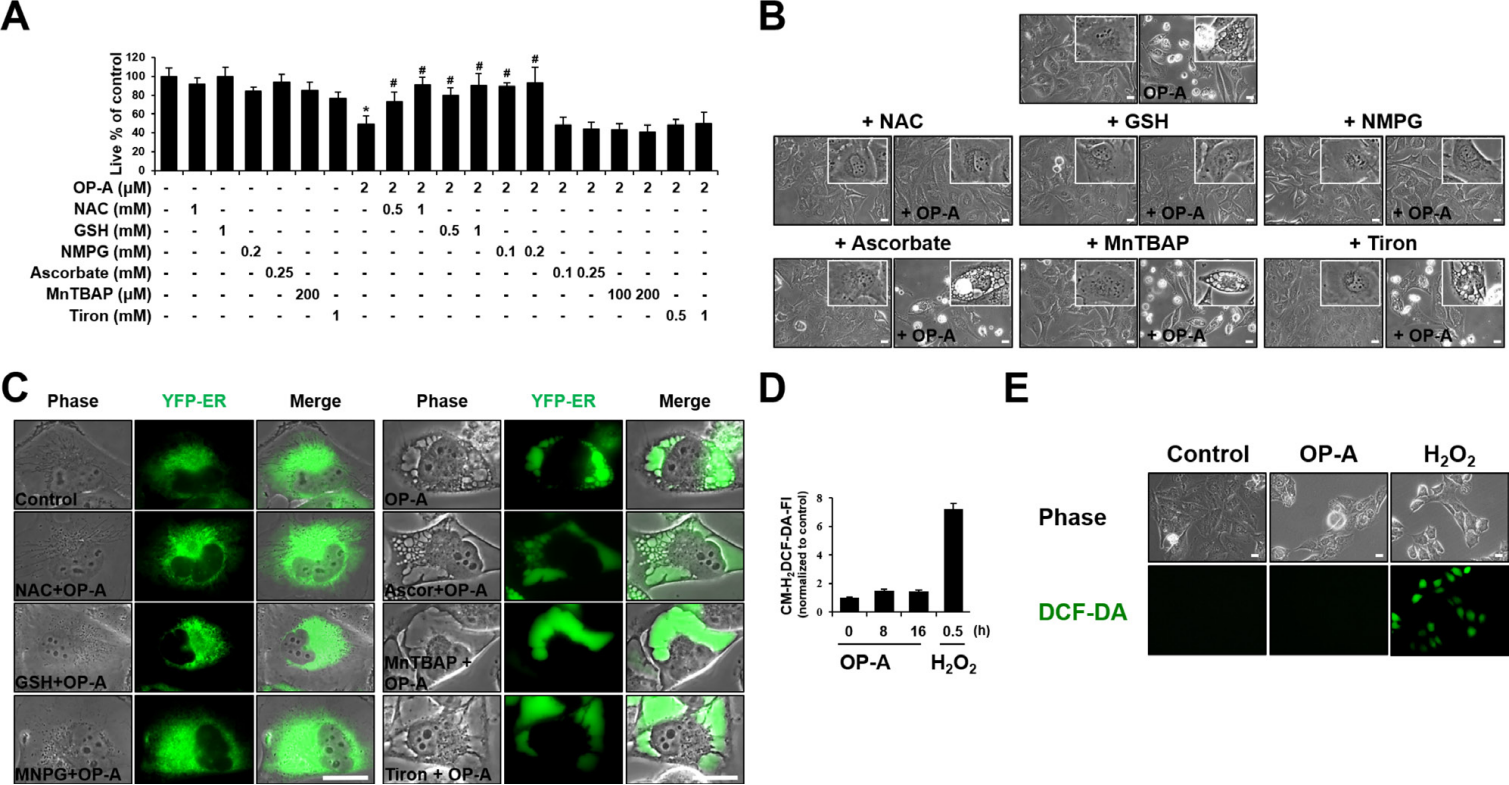
OP-A-induced cellular responses in T98G cells are effectively blocked by thiol antioxidants, but not by other ROS scavengers (**A**) Cellular viability was assessed using calcein-AM and EthD-1 in T98G cells pretreated with the indicated concentrations of various antioxidants and further treated with 2 µM OP-A for 24 h. Data represent the means ± SD (*n* = 7). One-way ANOVA and Bonferroni’s *post hoc* test. ^*^P < 0.001 vs. untreated control, ^#^*P* < 0.05 vs. OP-A treatment. (**B**, **C**) T98G (B) or YFP-ER (C) cells were pretreated with 1 mM NAC, 1 mM GSH, 0.2 mM NMPG, 0.25 mM ascorbic acid, 200 µM MnTBAP, or 1 mM Tiron and further treated with 2 µM OP-A for 16 h. Cells were observed under the phase-contrast microscope (B) or fluorescence microscopy (C). Bar 20 μm. (**D**) T98G cells were treated with 2 µM OP-A or 10 mM H_2_O_2_ for the indicated time points and then incubated with DCF-DA and subjected for flow cytometry. (**E**) T98G cells treated with 10 mM H_2_O_2_ for 30 min or 2 µM OP-A for 10 h were observed under the phase-contrast and fluorescence microscope. Bar, 20 μm.

**Figure 7:**
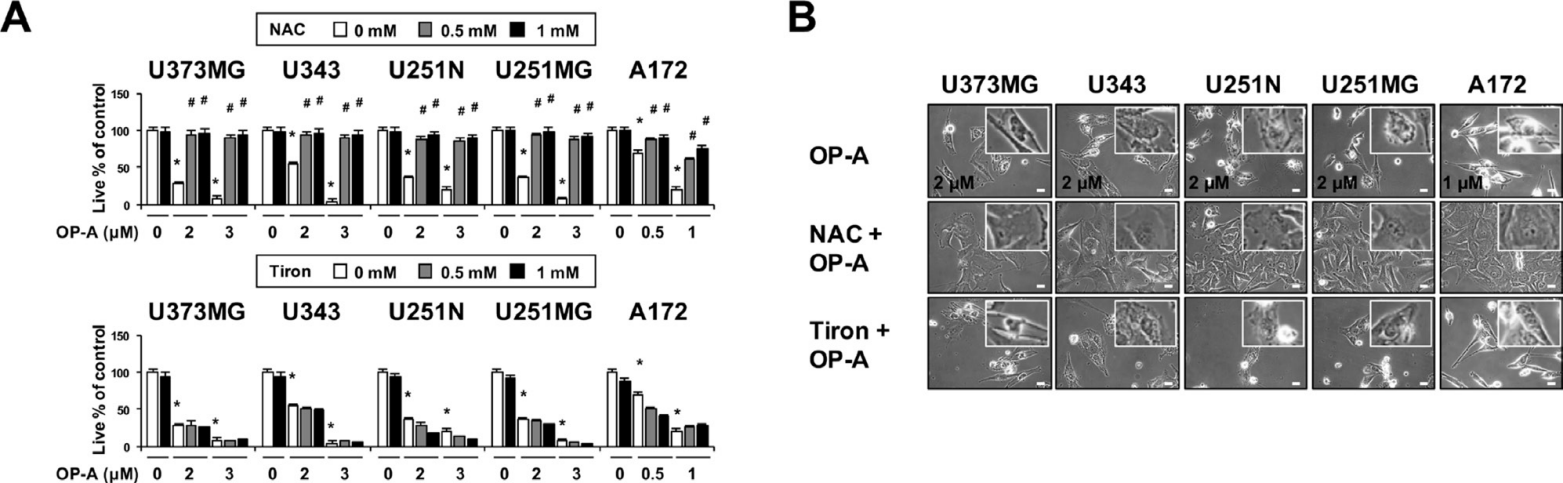
OP-A-induced cellular responses in various glioma cells are effectively blocked by NAC, but not by Tiron (**A**) Cells were pretreated with the indicated concentrations of NAC or Tiron and further treated with 2 µM OP-A for 24 h. Cellular viability was assessed using calcein-AM and EthD-1. Data represent the means ± SD (*n* = 7). One-way ANOVA and Bonferroni’s *post hoc* test. ^*^P < 0.001 vs. untreated control, ^#^*P* < 0.05 vs. OP-A treatment. (**B**) Cells pretreated with 1 mM NAC or 1 mM Tiron and further treated with the indicated concentrations of OP-A for 24 h were observed under the phase-contrast microscope. Bar, 20 μm.

Since we previously reported that the generation of ROS plays a critical role in curcumin-induced paraptosis [3], we tested the possible involvement of ROS in OP-A-induced paraptosis-like cell death. Interestingly, we found that pretreatment of T98G cells with various thiol-based antioxidants, including *N*-α–acetyl-L-cysteine (NAC), glutathione (GSH), and *N*-mercapto-propionyl-glycine (NMPG), effectively blocked OP-A-induced cell death (Figure 6A) as well as vacuolation derived from the ER (Figure 6B and 6C), but various non-thiol ROS scavengers, including ascorbic acid (Vitamin C), MnTBAP (Mn(III)tetrakis (4-benzoic acid) porphyrin) (a superoxide dismutase (SOD) mimetic), and Tiron (a radical scavenger), did not (Figure 6A–6C). Flow cytometry and fluorescence microscopy performed using CM-H_2_DCF-DA showed that, in contrast to H_2_O_2_, OP-A treatment did not noticeably increase ROS levels (Figure 6D and 6E). The ability of NAC, but not Tiron, to block OP-A-induced vacuolation and cell death was confirmed in various glioma cell lines (Figure 7A and 7B). These results suggest that OP-A demonstrates its anti-glioma activity via a thiol-dependent mechanism, rather than through ROS.

### Covalent modification of the free thiol groups on intracellular proteins may critically contribute to OP-A-mediated cytotoxicity

**Figure 8:**
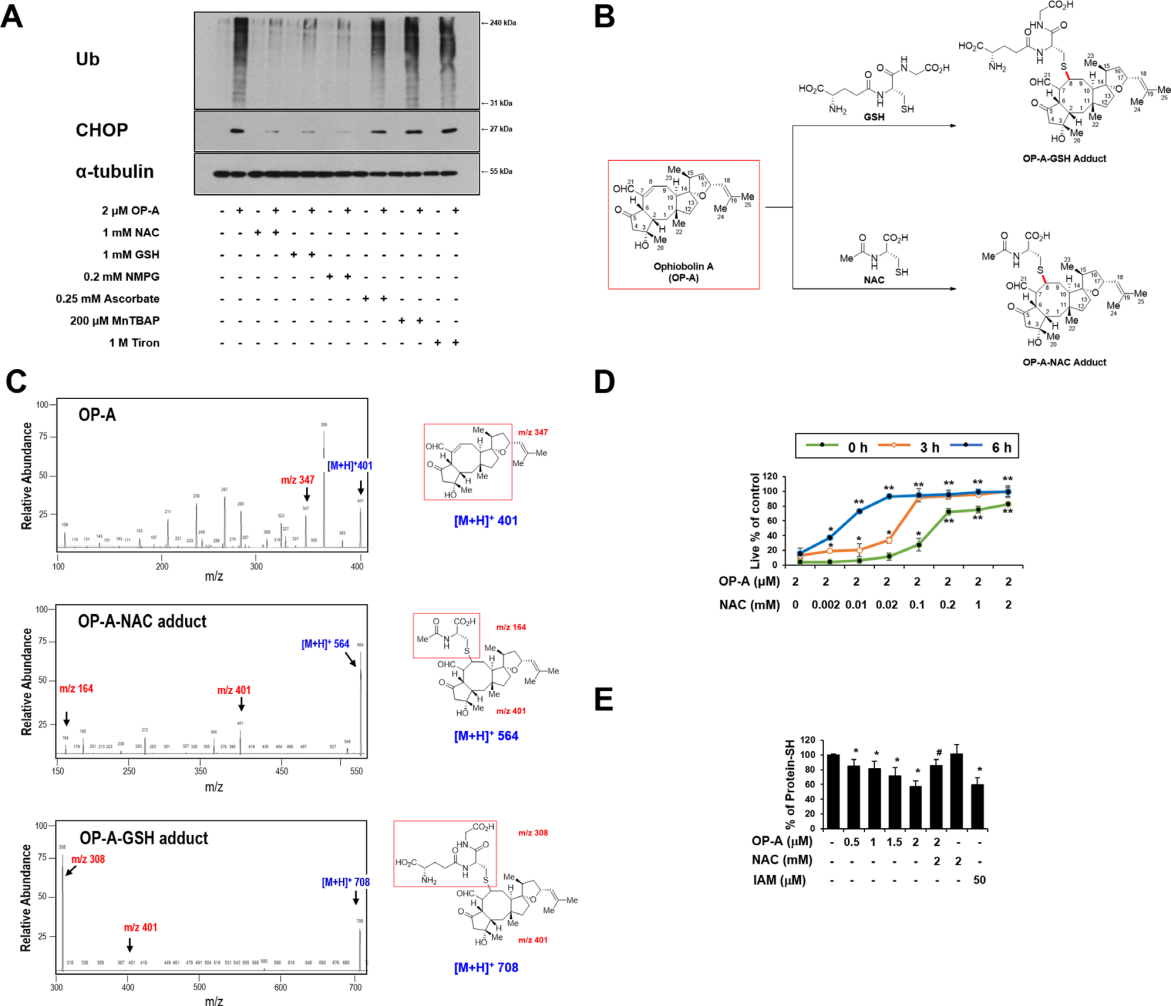
The thiol reactivity of OP-A is critical for its ability to induce paraptosis-like cell death in glioma cells (**A**) T98G cells were pretreated with the indicated concentrations of the respective antioxidant and further treated with 2 µM OP-A for 12 h. Cell extracts were subjected to Western blotting for the indicated proteins. (**B**) Proposed chemical structures of the OP-A-GSH and OP-A-NAC adducts. (**C**) Full-scan product ion scan spectra and the expected structures of OP-A, OP-A-NAC, and OP-A-GSH adducts formed upon Michael addition of NAC or GSH. The *m/z* values of the OP-A-NAC adduct represent NAC at 164, OP-A at 401, and the adduct form at 564. The *m/z* values of the OP-A-GSH adduct represent GSH at 308, OP-A at 401, and the adduct form at 708. (**D**) Increasing concentrations of NAC were pre-incubated with 2 µM OP-A in serum-free medium for the indicated time durations at room temperature, and these mixtures were used to treat T98G cells for 24 h. The relative cell viability was measured using calcein-AM and EthD-1. Data represent the means ± SD (*n* = 7). One-way ANOVA and Bonferroni’s *post hoc* test. ^*^P < 0.001, ^**^P < 0.0001 vs. OP-A-treated cells. (**E**) T98G cells were treated with the indicated concentrations of NAC and/or OP-A for 4 h. As a positive control to reduce intracellular protein-SH levels, IAM was used. T98G cells were treated with 50 µM IAM for 4 h. Protein-SH levels were measured using the dibromobimane assay, as described in the Materials and Methods. Data represent the means ± SD. Kruskal-Wallis test was performed followed by Dunn’s test. ^*^P < 0.001 vs. untreated control, ^#^*P* < 0.001 vs. OP-A treatment.

**Figure 9:**
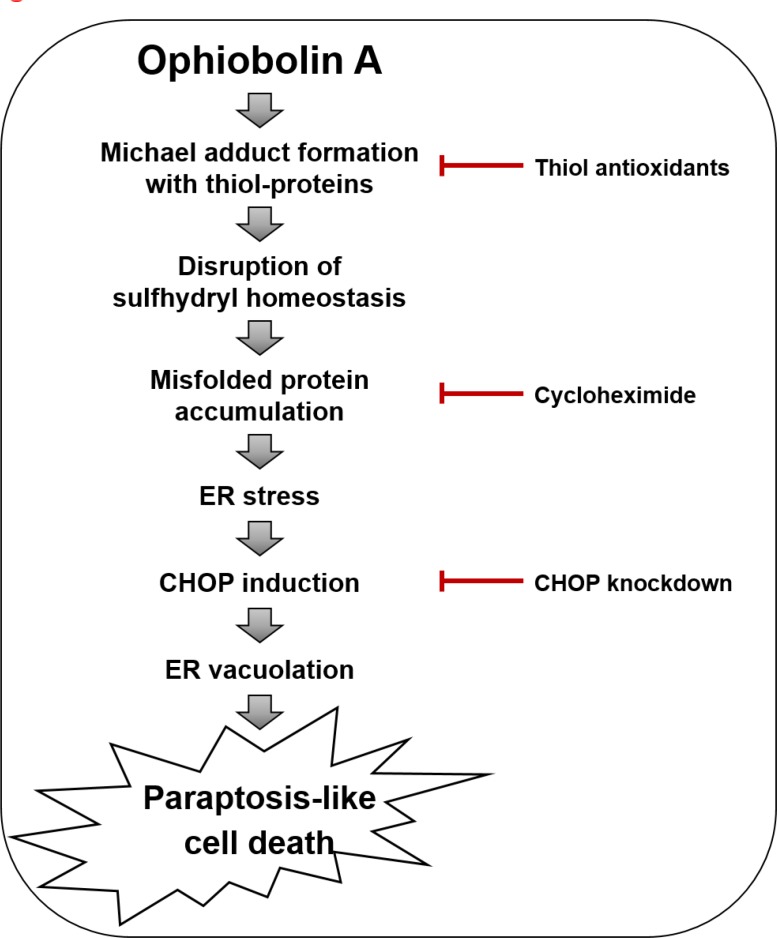
Hypothetical model for the underlying mechanism of OP-A-induced paraptosis-like cell death

When we further examined the effects of thiol antioxidants on OP-A-induced ER stress, we found that the OP-A-mediated accumulation of poly-ubiquitinated proteins and CHOP in T98G cells was effectively inhibited by various thiol antioxidants, but not by non-thiol antioxidants (Figure 8A), suggesting that thiol antioxidants may block the OP-A-induced cellular response at its initial step. We thus set out to examine whether the abilities of NAC and GSH to abrogate OP-A-mediated cytotoxicity reflected their direct interaction with OP-A. In structural terms, we speculated that the highly electrophilic character of the α,β-unsaturated ketone substructure of OP-A at C8 might allow it to react with the thiol groups of NAC and GSH to form covalent Michael adducts, such as NAC-OP-A and GSH-OP-A conjugates (Figure 8B). To test this hypothesis, we incubated 100 µM OP-A with excess NAC (50 mM) for 3 h and then analyzed the reaction mixture by LC-MS/MS. MS scan revealed a peak at 564 ([M+H]^+^) corresponding to the molecular weight of the Michael addition product, and its representative fragmentation pattern in the product ion scan showed peaks at 401 ([M+H]^+^) and 164 ([M+H]^+^), which corresponded to the molecular weights of OP-A and NAC, respectively (Figure 8C). The chromatogram of LC-MS/MS analysis showed the OP-A-NAC adduct peak, which was monitored by mass transition from 564 to 401, was eluted at 2.1 min (Supplementary Figure 6), confirming the formation of an adduct between OP-A and NAC. Comparable results were obtained when we analyzed the formation of an adduct between OP-A and GSH (Figure 8C and Supplementary Figure 6). To investigate the effect of the interaction between OP-A and NAC on OP-A-mediated cytotoxicity, we pre-incubated 2 µM OP-A with different doses of NAC in serum-free medium at room temperature for 24 h (to allow the formation of chemical adducts) and then treated T98G cells with each OP-A/NAC mixture. At a given dose of NAC, cells treated with OP-A and NAC that had undergone the prolonged pre-incubation showed far less OP-A-mediated cytotoxicity than those subjected to simultaneous treatment; moreover, pre-incubation required a lower concentration of NAC to block OP-A-mediated cell death to the same extent, compared to simultaneous treatment (Figure 8D). These results strongly suggest that NAC blocks OP-A cytotoxicity by eliminating its ability to form Michael adducts, particularly with the nucleophilic thiol groups of intracellular proteins. To further test whether OP-A directly reacts with the free thiol residues of the proteins, we performed dibromobimane (dBrB) assay, which is based on the ability of dBrB to react with free reduced thiols and generate a highly fluorescent protein-dBrB adduct [24, 25]. When we used iodoacetamide (IAM), an alkylating agent that reacts with protein-SH groups to form stable S-carboxyaminodomethyl-cysteine adducts [25, 26] as a positive control, IAM treatment effectively reduced free protein-SH levels in T98G cells (Figure 8E). OP-A treatment also decreased the protein-SH levels dose-dependently, whereas combined treatment with NAC and OP-A did not (Figure 8E). When extracts of OP-A-treated T98G cells were incubated with dithiothreitol (DTT), which converts disulfide bonds into free protein-SH groups, we still observed DTT-resistant protein-SH depletion by OP-A (Supplementary Figure 7), suggesting the formation of the stable adducts between OP-A and the thiol-containing proteins. Collectively, our results indicate that OP-A may covalently modify the free thiol groups of intracellular proteins, thereby disrupting intracellular thiol homeostasis. The formation of Michael adducts between OP-A and the cysteine residues of intracellular proteins may decrease proper disulfide bond formation during protein folding, leading to ER stress, ER dilation, and paraptosis-like cell death (Figure 9).

## DISCUSSION

OP-A, a natural compound that a certain fungus generates to attack plant cells [15], was known to act against apoptosis-resistant GBM cells by inducing paraptosis-like cell death [10], but the underlying mechanism was not clearly understood. In the present study, we confirm that OP-A induces paraptosis-like cell death as a main cell death mode in various glioma cells. Mechanistically, we show that ER stress is the critical underlying mechanism of OP-A-mediated anti-glioma activity. The ER is the subcellular organelle in which secretory and membrane proteins are folded, stabilized by disulfide bonds, post-translationally modified, oligomerized, and ultimately exported. The unfolded protein response (UPR) is triggered by the accumulation of misfolded proteins in the ER lumen and attempts to restore homeostasis; however, when early UPR fails to restore ER functions, terminal UPR triggers ER stress-induced cell death [27]. Supporting this idea, our Western blotting on the UPR components showed that OP-A treatment only transiently upregulated the phosphorylation levels of PERK and eIF2α and the expression of ATF4, but CHOP upregulation was highly sustained. When we examined the functional significance of CHOP upregulation in OP-A-induced cellular responses, CHOP knockdown employing both siRNA and shRNA effectively blocked OP-A-induced cell death as well as cell vacuolation (Figure 5 and Supplementary Figure 3). These results suggest that CHOP plays a critical role in OP-A-induced paraptosis-like cell death. In contrast the effect of CHOP knockdown, the siRNA-mediated knockdown of ATF4 did not affect OP-A-induced vacuolation or cell death (Supplementary Figure 4). Furthermore, both siRNA-mediated knockdown of PERK and inhibition of PERK activity using its specific inhibitor GSK2656157 had no effect on OP-A-induced paraptosis-like cell death (Supplementary Figure 5). These results suggest that, unlike CHOP, ATF4 and PERK are not critically involved in OP-A-induced paraptosis-like cell death.

In terms of the potential action mechanism of OP-A, Dasari *et al.* [21] proposed that its toxicity relies on the condensation of its C5,C21-dicarbonyl moiety with the primary amines of intracellular proteins, leading to their covalent modification. Chidley *et al.* [28] recently identified phosphatidylethanolamine (PE) as a molecular target of OP-A and proposed that the formation of PE-OP-A adducts directly causes OP-A cytotoxicity through membrane destabilization. As OP-A possesses a highly electrophilic α,β-unsaturated ketone substructure, we herein propose that that the ability of OP-A to function as an electrophile and form adducts with thiol-containing proteins is critical for its anti-cancer effects. This is supported by the following novel findings presented herein: (a) OP-A undergoes chemical reactions with NAC and GSH to generate NAC-OP-A and GSH-OP-A adducts, respectively, *in vitro*. (b) Pre-incubation of NAC with OP-A ameliorates the cytotoxicity of OP-A toward glioma cells. (c) OP-A-induced cell death is blocked by various thiol antioxidants, but not by other ROS scavengers, in many GBM cells and cancer cells of different tissue origins. (d) Pretreatment with thiol antioxidants completely blocks OP-A-induced all the other cellular responses in cancer cells. (e) OP-A treatment reduces the intracellular sulfhydryl protein (protein-SH) content in T98G cells. Since NAC and GSH are thiol nucleophiles (in addition to being antioxidants), we speculated that the α,β-unsaturated ketone substructure of OP-A may be susceptible to conjugation with NAC or GSH to form Michael adducts and thus, they may protect cells against OP-A-induced cytotoxicity by blocking the ability of OP-A to form Michael adducts with intracellular thiol nucleophiles. Furthermore, the ability of OP-A to reduce the content of intracellular free sulfhydryl proteins suggests that the formation of Michael adducts with intracellular nucleophiles may be critically contribute to OP-A-induced toxicity, perhaps by disrupting proper disulfide bond formation to cause protein misfolding and the subsequent accumulation of misfolded proteins within the ER. The OP-A-induced formation of adducts among thiol-containing folding-chaperone proteins may further aggravate this misfolding. Interestingly, CHOP, which we found to be critical for OP-A-induced paraptosis-like cell death from its knockdown experiment, lacks a cysteine residue, and thus may not undergo OP-A-mediated adduct formation. CHOP is a stress-inducible transcription factor that mediates cell death and is critical for the determination of cellular fate [29]. Han *et al.* [30] recently reported that CHOP-mediated transcriptional regulation increases protein synthesis, critically contributing to ER-stress-induced cell death. Therefore, further work is warranted to determine whether the induction of CHOP observed in the present work contributes to ER dilation by altering the transcriptional control of specific protein(s) and/or causing a global increase in protein synthesis. Compared to mature proteins, newly synthesized proteins may be more sensitive to covalent modification by OP-A. This might explain why CHX pretreatment protect all of the tested glioma cell lines from OP-A-induced ER-derived vacuolation and cell death, in that it might reduce the overall load of misfolded proteins in the ER. Previously, Mimnaugh *et al.* [22] proposed that misfolded proteins overloaded within the ER lumen could exert an osmotic force to draw water from the cytoplasm and distend the ER lumen. Therefore, the OP-A-induced accumulation of misfolded proteins could trigger ER-derived cytoplasmic vacuolation, leading to structural/functional defects in the ER and subsequent paraptosis-like cell death.

Morrison *et al.* [19] very recently showed that OP-A induces different mechanisms of cell death in mammalian cells depending on the cancer cell origin. When we first tested the cell-killing effects of OP-A in various cancer cell lines of different origins, including Huh-7 (hepatocellular carcinoma), U2OS (osteosarcoma), and MDA-MB 468 (breast cancer), HeLa (cervical cancer), and BxPC-3 (pancreatic cancer) cells, OP-A had varied but dose-dependent cytotoxic effects on these cancer cells (Supplementary Figure 8A). When we further examined the major cell death mode(s) induced by OP-A using z-VAD-fmk, necrostatin-1, and CHX, to block apoptosis, necroptosis, and paraptosis, respectively, pretreatment with CHX effectively recovered cell viability in these cancer cells treated with OP-A (Supplementary Figure 8B). However interestingly, OP-A-induced cell death in Huh-7, U2OS, and MDA-MB 468 cells was partially but significantly inhibited by either z-VAD-fmk or necrostatin-1 pretreatment. In contrast, OP-A-induced cell death in HeLa and BxPC-3 cells was significantly attenuated by necrostatin-1, but not by z-VAD-fmk pretreatment. These results suggest that OP-A can induce mixed types of cell death, including paraptosis, apoptosis and/or necrosis, possibly depending on the genetic background of the tumor cell line. In various GBM cell lines, however, OP-A mainly induced paraptosis-like cell death without the involvement of apoptosis or necroptosis. These results indicate that although GBM cells are resistant to the induction of apoptosis or necroptosis, they may still be vulnerable to the induction of paraptosis. Therefore, strategies to induce paraptosis may provide a way to effectively treat human GBM, a deadly malignancy that is resistant to various anti-cancer treatments.

Dasari *et al.* [21] reported that OP-A treatment significantly enhanced survival and reduced tumor growth in mice bearing orthotopic U251-LUC tumors. In addition, Bury *et al.* showed that OP-A significantly increased survival in the B16F10 mouse melanoma model with lung peudometastases [16]. To support the potential application of OP-A in the clinic, however, further studies may be required to confirm its safety and efficacy in cancer therapy. When we tested the effect of OP-A on normal cells, OP-A did not significantly affect the viability of astrocytes and had minimal effects on normal liver cells (Supplementary Figure 9A). Furthermore, astrocytes and Chang cells treated with 2 µM OP-A did not noticeably induce vacuolation (Supplementary Figure 9B). These results suggest that OP-A treatment may induce paraptosis-like cell death preferentially in glioma and hepatoma cells, sparing their counterpart normal cells. Interestingly, we found that OP-A-mediated cytotoxicity was almost completely blocked by NAC, but not by Tiron, in all of the tested cancer cell lines (Figure 6A, 7A, and Supplementary Figure 8C), irrespective of the OP-A-induced cell death mode(s). This suggests that the ability of OP-A to covalently modify free sulfhydryl groups on proteins and thereby disrupt thiol homeostasis may be a common mechanism through which this agent exerts its anti-cancer effects.

In summary, we herein show for the first time that Michael adduct formation and the resulting ER stress critically contribute to OP-A-induced toxicity in cancer cells. The ability of OP-A to disrupt intracellular sulfhydryl proteostasis, leading to ER stress and dilation, may represent an effective therapeutic strategy against human glioblastoma.

## MATERIALS AND METHODS

### Chemicals

All chemicals were purchased from Sigma-Aldrich (St Louis, MO, USA) unless indicated otherwise. Ophiobolin A (OP-A) was from Adipogen (San Diego, CA, USA). Cycloheximide (CHX), MitoTracker-Red (MTR), calcein-acetoxymethyl ester (calcein-AM), ethidium homodimer-1 (EthD-1), and 4′,6-diamidino-2-phenylindole (DAPI), CM-H_2_DCF-DA was from Molecular probe (Eugene, OR, USA). Benzyloxy-carbonyl-Val-Ala-Asp-(OMe)-fluoromethyl ketone (z-VAD-fmk) was from R&D systems (Minneapolis, MN, USA). Tiron was from Fluka (Schwerte, Deutschland). Mn(III)tetrakis (4-benzoic acid) porphyrin (MnTBAP) was from Calbiochem (EDM Millipore Corp., Billerica, MA, USA).

### Cell culture

T98G, U251N, and A172 cells were purchased from ATCC (Manassas, VA, USA). U373MG cells were purchased from Sigma-Aldrich. U343 and U251MG cells were purchased from CLS (Berlin, Germany). Cells were cultured in Dulbecco’s Modified Eagle’s Medium (DMEM) (Gibco-BRL) supplemented with 10% fetal bovine serum (FBS) (Gibco-BRL, Waltham, MA, USA) and 1X antibiotics (Gibco-BRL). Cell lines were used at low passage numbers from primary stocks and were routinely tested for mycoplasma.

### Determination of cellular viability using calcein-AM and EthD-1 (Live/Dead assay)

Cell viability using calcein-AM and EthD-1 was assessed as described previously [3]. Only exclusively green cells were counted as live because bicolored (green and red) cells cannot be unambiguously assigned to live or dead groups. The percentage of live cells was normalized to that of untreated control cells (100%).

### Examination of the stable cell lines expressing the fluorescence specifically in the endoplasmic reticulum

The stable T98G sublines expressing the fluorescence specifically in the ER were established by transfection of T98G cells with the pEYFP-ER vector (Clontech, Mountain View, CA, USA) and selection with fresh medium containing 500 µg/mL G418 (Calbiochem, Darmstadt, Germany).

### Western blotting

Western blot analysis was performed as described previously [3] using the following antibodies: PARP (Abcam, Cambridge, MA, USA); caspase-3 (Stressgen, Ann Arbor, MI, USA); ubiquitin, ATF4, and α-tubulin (Santa Cruz biotechnology, Santa Cruz, CA, USA); total eIF2α, phosphor-eIF2α, IRE1α, PERK, and CHOP (Cell Signaling Technology, Beverly, MA, USA); Grp78 (Sigma-Aldrich); COX II (Invitrogen, Carlsbad, CA, USA); PDI (Enzo Life Sciences, Farmingdale, NY, USA); rabbit IgG HRP, mouse IgG HRP, and goat IgG HRP (Molecular probe, Eugene, OR, USA).

### Immunocytochemistry

Immunocytochemistry was performed as described previously [5] using the following antibodies: COX II (Invitrogen), PDI (Enzo Life Sciences), CHOP (Cell Signaling Technology), and ubiquitin (Santa Cruz biotechnology).

### Measurement of reactive oxygen species (ROS) production

Cells treated with OP-A or H_2_O_2_ were incubated with 5 μM of H_2_DCF-DA for 30 min in the dark at 37°C. After washing with Hank’s Buffered Salt Solution (HBSS) containing Ca^2+^ and Mg^2+^, cells were further processed for fluorescence activated cell sorting (FACS) analysis using a FACScan flow cytometer system (BD Biosciences, San Jose, CA) or fluorescence microscopy (Axiovert 200M, Carl Zeiss, Germany).

### Gene silencing of CHOP

The siRNA duplexes were purchased from Invitrogen (Carlsbad, CA, USA) with the sequence 5′-GAGCUCUGAUUGACCGAAUGGUGAA-3′ and as the control, Negative Universal Control (Invitrogen) was used. Transfection was performed using Lipofectamine RNAiMAX (Invitrogen, Carlsbad, CA). To confirm successful siRNA-mediated knockdown, Western blotting of CHOP was performed.

### *In vitro* reactions of OP-A with GSH or NAC, and LC-MS/MS analysis

OP-A was adjusted to 100 µM in 1 mL methanol and then mixed with 1 mL of either 50 mM GSH or 50 mM NAC. After 3 h incubation at 40°C, the reaction was quenched by the addition of 20 fold ice-cold methanol. For monitoring of OP-A alone and its formation of adducts with NAC or GSH, 1 μL aliquot of the mixture was directly injected into an Agilent 6470 Triple Quad LC-MS/MS system (Agilent, Wilmington, DE, USA) coupled to an Agilent 1260 HPLC system. Chromatographic separation was achieved using a Synergi Polar RP (4 µm, 2.0 mm i.d. × 150 mm, Phenomenex, Torrance, CA, USA).

### Detoxification of OP-A with N-acetylcysteine (NAC)

To test the detoxification of OP-A by NAC, aliquots of serum-free DMEM containing 2 µM OP-A and different concentrations of NAC were pre-incubated at room temperature for the indicated time points, and then incubated with T98G cell cultures for 24 h. To examine the effect of simultaneous treatment of OP-A and NAC, cells were treated with 2 µM OP-A and increasing concentrations of NAC without pre-incubation. Subsequently, cell viability was assessed using calcein-AM and EthD-1.

### Fluorescence labeling of protein thiol groups (dibromobimane assay)

The depletion of protein thiol groups (protein-SH) following OP-A exposure was measured using the previously described dibromobimane (dBrB) assays [24, 25] with minor modifications. T98G cells plated in 12-well plates were treated with various concentrations of OP-A, harvested, resuspended in PBS, and sonicated. A part of samples was used for the measurement of the protein concentration using Lowry-based assay and the rest of samples were immediately reacted with 1.5 N perchloric acid and incubated for 5 minutes on ice to precipitate the proteins. The samples were centrifuged at 14,000 × g for 10 minutes, and the pelleted proteins were solubilized with 0.1 M NaOH and neutralized using 0.5 M Tris-HCl. The prepared proteins (5 μg) were mixed with 40 µM dibromobimane and incubated for 40 minutes at 37°C. Dibromobimane-bound protein-SH groups were measured in a fluorescence multiplate reader (Synergy H1 Hybrid Multi-Mode reader, BioTek, Winooski, VT, USA) at Ex/Em 393/477 nm. Fluorescence was normalized by the total protein levels and expressed as % of protein-SH levels compared to that from the untreated group.

### Statistical analysis

Statistical analysis was performed using the Prism 5.03 software (GraphPad Prison, Lajolla CA, USA) was used. Unless otherwise specified, each experiment was repeated at least three times. Normality of data was assessed by Kolmogorov-Smirnov testes and equal variance using Bartlett’s test. For normal distribution, statistical differences were determined using an analysis of variance (ANOVA) followed by Bonferroni multiple comparison test. If the data were not normally distributed, Kruskal-Wallis test was performed followed by Dunn’s test. *p* < 0.0001 was considered statistically significant.

## SUPPLEMENTARY MATERIALS FIGURES


