# Supplementary material for: Ophiobolin A kills human glioblastoma cells by inducing endoplasmic reticulum stress via disruption of thiol proteostasis
Source: Oncotarget. 2017 Nov 20;8(63):106740–52. doi: 10.18632/oncotarget.22537 (PMC5739770; doi:10.18632/oncotarget.22537)
Supplement: Supplementary file 1 [file oncotarget-08-106740-s001.pdf]

Ophiobolin A kills human glioblastoma cells by inducing endoplasmic reticulum stress via disruption of thiol proteostasis

SUPPLEMENTARY MATERIALS

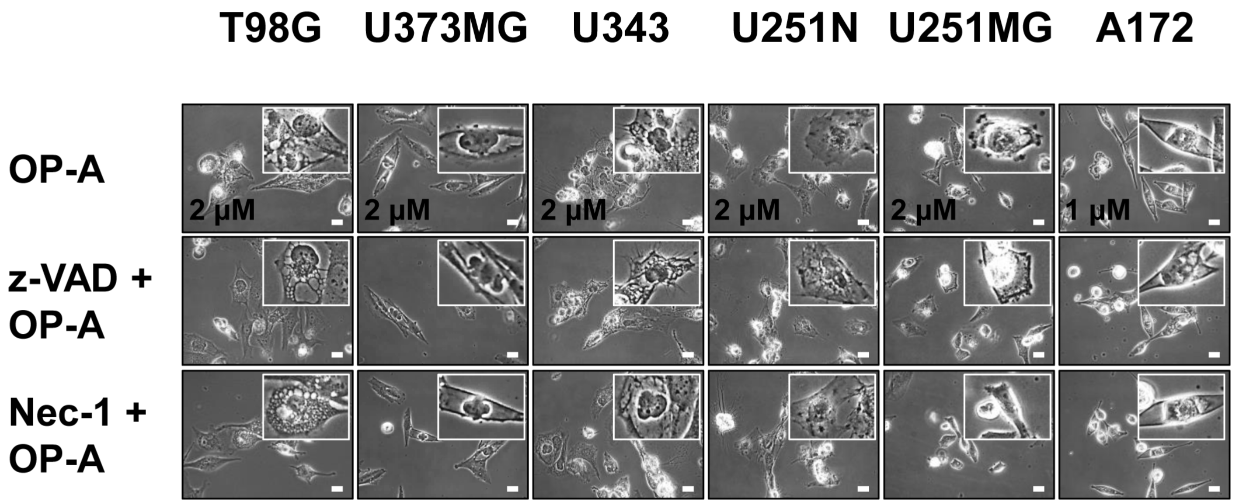

Supplementary Figure 1: OP-A-induced vacuolation is not affected by pretreatment with z-VAD-fmk or necrostatin-1. Cells were pretreated with 20 µM z-VAD or 80 µM necrostatin-1 for 30 min and further treated with 2 µM OP-A (1 µM OP-A in A172 cells) for 24 h and observed under the phase-contrast microscope. Bar, 20 µm.

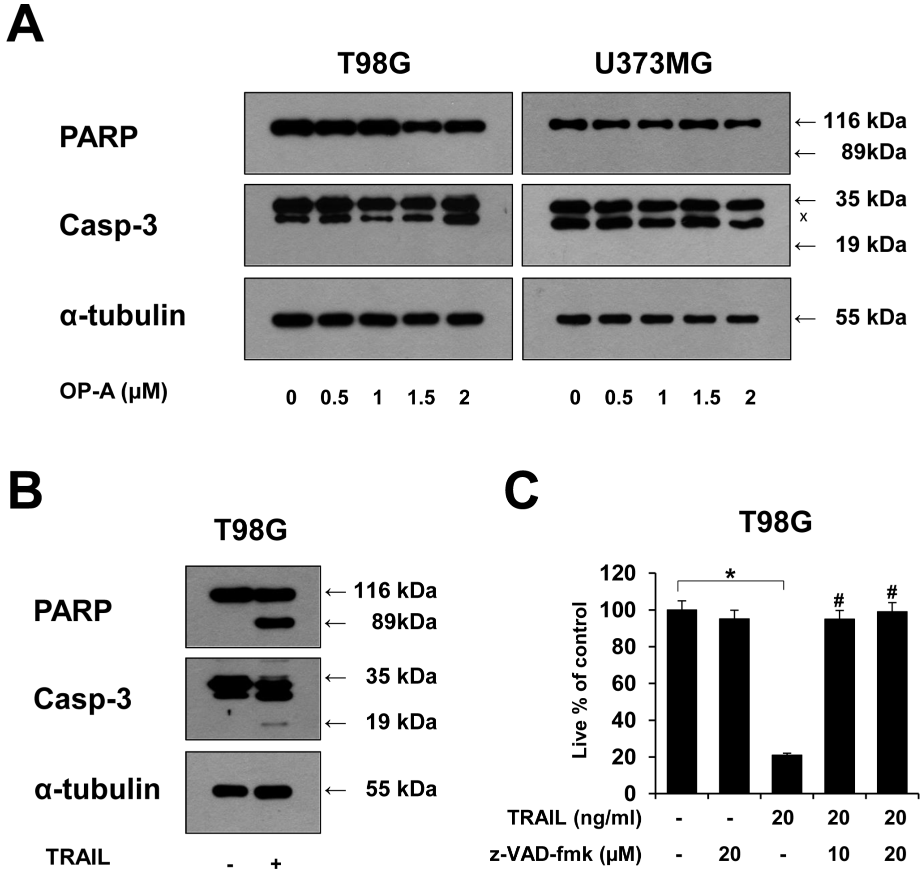

Supplementary Figure 2: Caspase-mediated apoptosis is not involved in OP-A-induced cell death of glioma cells. (**A**) Cells were treated with the indicated concentrations of OP-A for 24 h. Cell extracts were prepared from the treated cells and Western blotting of PARP and caspase-3 was performed α-tubulin was used as a loading control in Western blots. (**B**) Cell extracts were prepared from T98G cells untreated or treated with 20 mg/ml TRAIL (the nontagged 19 kDa protein, amino acids 114-281, KOMA Biotech (Seoul, South Korea)) for 24 h and Western blotting of PARP and caspase-3 was performed. (**C**) T98G cells pretreated with 20 μM z-VAD-fmk were further treated with 2 μM OP-A for 24 h. Cellular viability was assessed using calcein-AM and EthD-1. Data represent the means ± SD ($n = 7$). One-way ANOVA and Bonferroni's *post hoc* test. $^{*}P <$ 0.001 vs. untreated control, $^{\#}P <$ 0.05 vs. OP-A treatment.

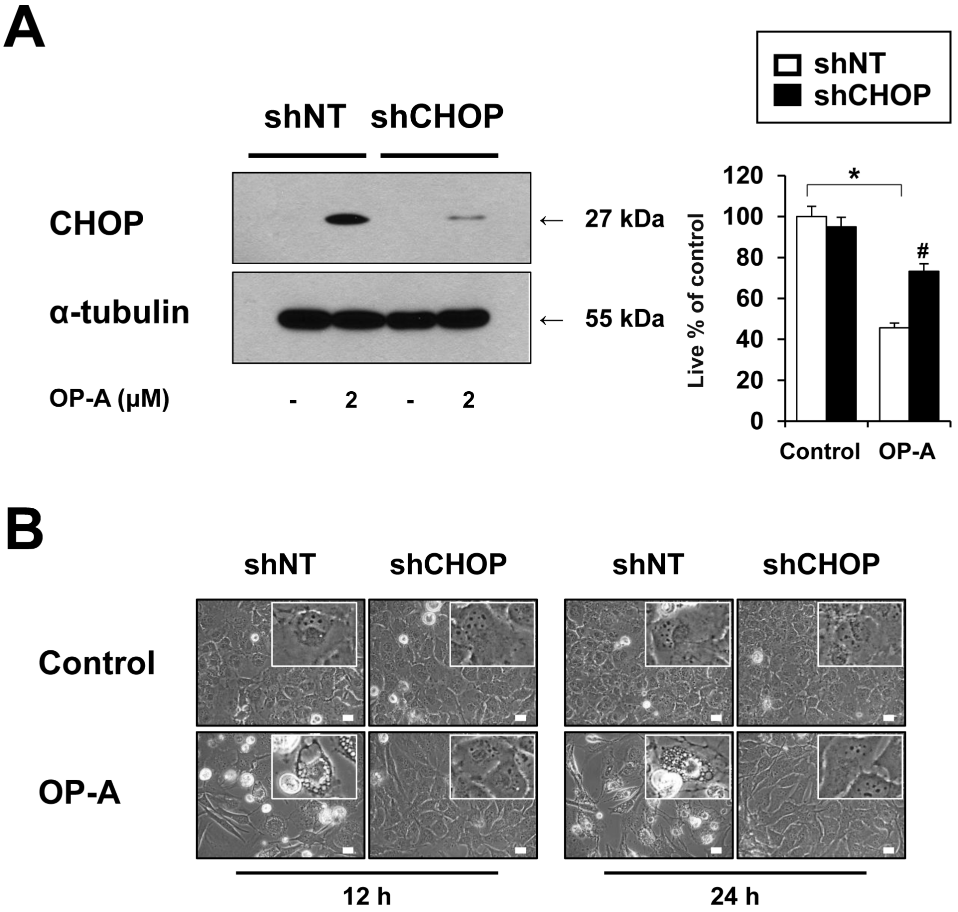

Supplementary Figure 3: shRNA-mediated knockdown of CHOP attenuates OP-A-induced paraptosis-like cell death. For the knockdown experiments using CHOP-targeting shRNA, HEK293TN cells were transfected with the plasmid containing the non-targeting shRNA (SHC002V, Sigma-Aldrich) or the plasmid containing CHOP-targeting shRNA (TRCN0000364328, Sigma-Aldrich), together with pMD2.G (the envelope plasmid) and pPsAX2.0 plasmid (the packaging plasmid) using TransIT-2020 transfection reagents (Mirus Bio LLC, Madison, WI, USA) according to the manufacturer's instructions. After 48 h of lentiviral particle production, medium was harvested and filtered. T98G cells were infected with the filtered lentiviral medium encoding non-targeting (NT) shCHOP or CHOP shRNA, which was supplemented with 10 μg/ml polybrene. (**A**) Infected cells were further treated with 2 μM OP-A for 24 h. Western blotting of CHOP and α-tubulin was performed to confirm CHOP knockdown (*left*). Cellular viability was assessed using calcein-AM and EthD-1 (*right*). Data represent the means ± SD (n = 7). One-way ANOVA and Bonferroni's *post hoc* test. $^{*}P <$ 0.001 vs. untreated control, $^{\#}P <$ 0.05 vs. OP-A treatment. (**B**) Infected cells were further treated with 2 μM OP-A for the indicated time points and observed under the phase-contrast microscope. Bar, 20 μm.

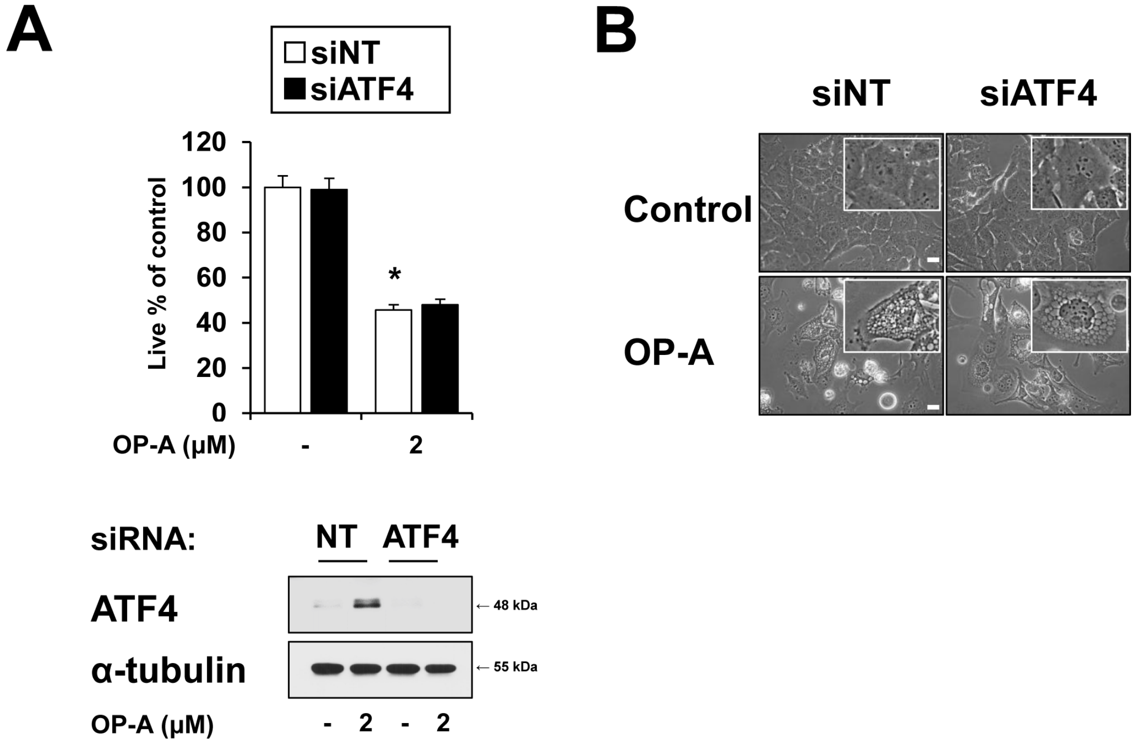

Supplementary Figure 4: ATF4 upregulation may not be critically involved in OP-A-induced paraptosis-like cell death. (**A**) The siRNA duplexes of ATF4 were purchased from Santa Cruz (sc-35112) and as the control, Negative Universal Control (Invitrogen) was used. T98G cells transfected with the non-targeting siRNA (siNT) or ATF4 siRNA were further treated with 2 μM OP-A for 24 h. Cellular viability was assessed using calcein-AM and EthD-1 (upper panel). Data represent the means ± SD (n = 7). One-way ANOVA and Bonferroni's *post hoc* test. $^{*}P <$ 0.001 vs. untreated control. ATF4 knockdown was confirmed by western blotting (*lower panel*). (**B**) Treated cells were observed under the phase-contrast microscope. Bar, 20 μm.

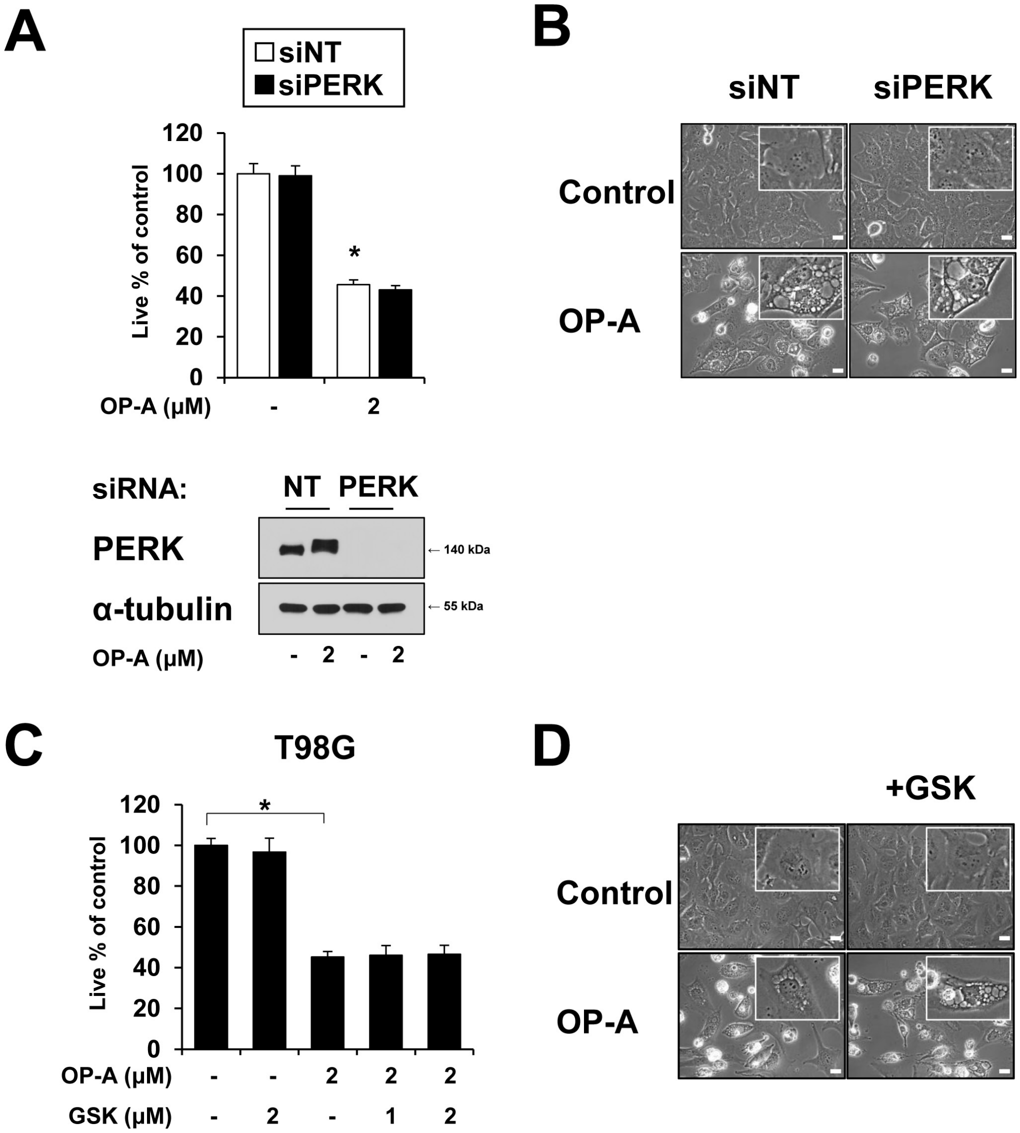

Supplementary Figure 5: PERK may not be critically involved in OP-A-induced paraptosis-like cell death. (**A**) The siRNA duplexes of PERK were purchased from Santa Cruz (sc-36213) and as the control, Negative Universal Control (Invitrogen) was used. T98G cells transfected with the non-targeting siRNA (siNT) or PERK siRNA were further treated with 2 μM OP-A for 24 h. Cellular viability was assessed using calcein-AM and EthD-1 (*upper panel*). Data represent the means ± SD ($n = 7$). One-way ANOVA and Bonferroni's *post hoc* test. $^{*}P < 0.001$ vs. untreated control, PERK knockdown was confirmed by western blotting (*lower panel*). (**B**) Treated cells were observed under the phase-contrast microscope. Bar, 20 μm. (**C**) T98G cells were pretreated with or without GSK2656157 and further treated with 2 μM OP-A for 24 h. Cellular viability was assessed using calcein-AM and EthD-1. Data represent the means ± SD ($n = 7$). One-way ANOVA and Bonferroni's *post hoc* test. $^{*}P < 0.001$ vs. untreated control. (**D**) Treated cells were observed under the phase-contrast microscope. Bar, 20 μm.

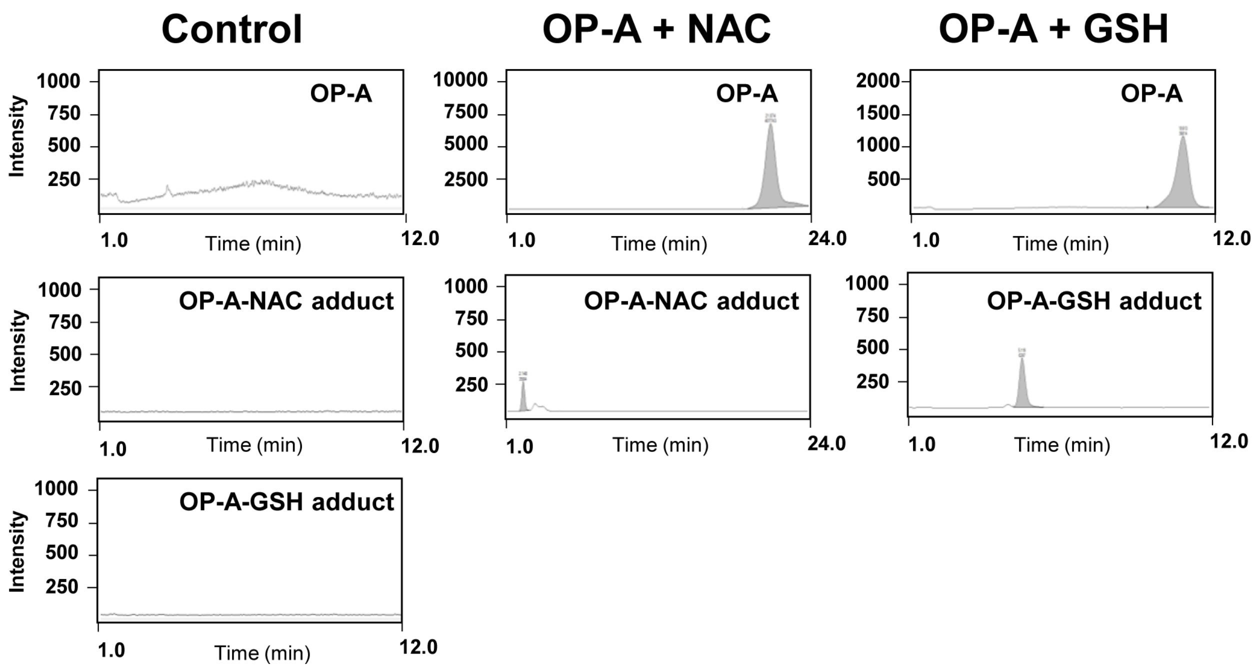

Supplementary Figure 6: Confirmation of OP-A-NAC and OP-A-GSH adduct by the LC-MS/MS analysis with a MRM mode. Equal volume of 100 μM OP-A and 50 mM NAC or 50 mM GSH in methanol were mixed and analyzed by LC-MS/MS after 3 h incubation at 40ºC. The analytes were eluted with a mobile phase consisting of 0.1% formic acid in water: 0.1% formic acid in methanol at a ratio of 35:65 (v/v) and a flow rate of 0.25 mL/min for OP-A-NAC adducts, or a ratio of 27:73 (v/v) and a flow rate of 0.2 mL/min for OP-A-GSH adducts. Peak identification was carried out using multiple reaction monitoring (MRM) in the positive ionization mode at m/z 401 à 347 for OP-A, m/z 564 à 401 for OP-A-NAC adducts and m/z 708 à 308 for OP-A-GSH adducts. Representative chromatograms of control, OP-A-NAC adduct formation, and OP-A-GSH adduct formation are shown.

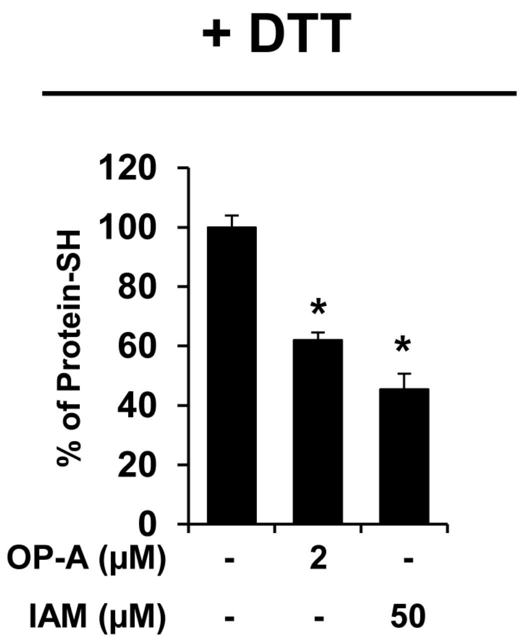

Supplementary Figure 7: DTT incubation does not restore intracellular protein-SH levels depleted by OP-A. T98G cells were treated with 2 μM OP-A or 50 μM IAM for 12 h, harvested, sonicated and incubated with 10 mM DTT for 30 min at room temperature. The excess of DTT was removed by dialysis, and protein-SH groups were analyzed as described in the Materials and Methods. Data represent the means ± SD. Kruskal-Wallis test was performed followed by Dunn's test. $^{*}P < 0.001$ vs. untreated control.

A

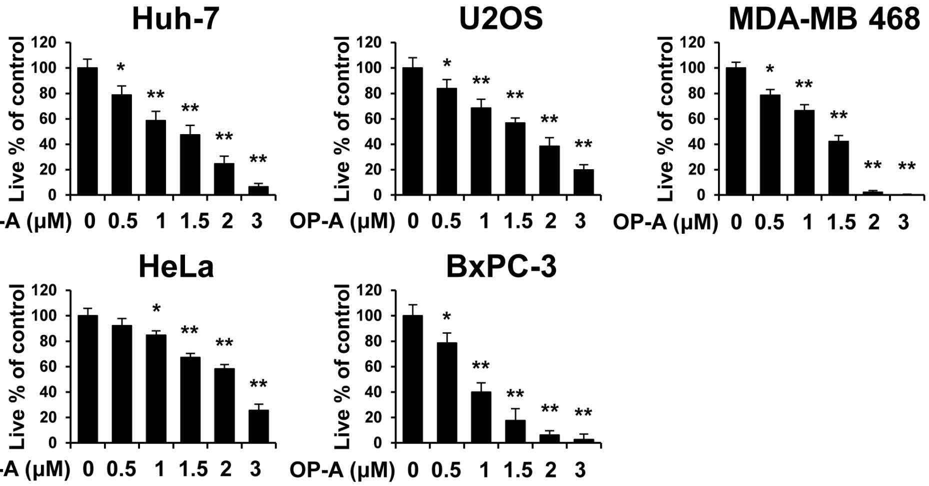

Cancer cell lines	Huh-7	U2OS	MDA-MB 468	HeLa	BxPC-3
IC_{50} (μM)	1.420	1.749	1.287	2.189	0.843

B

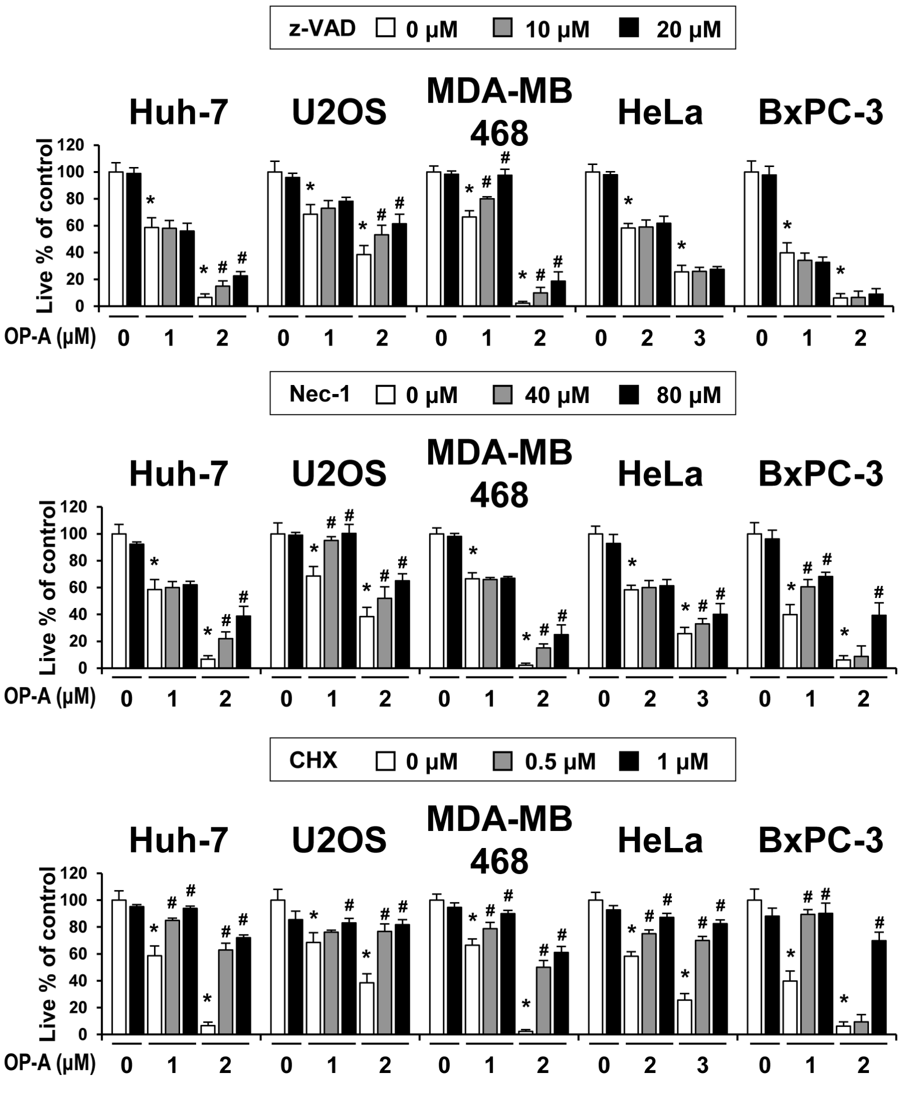

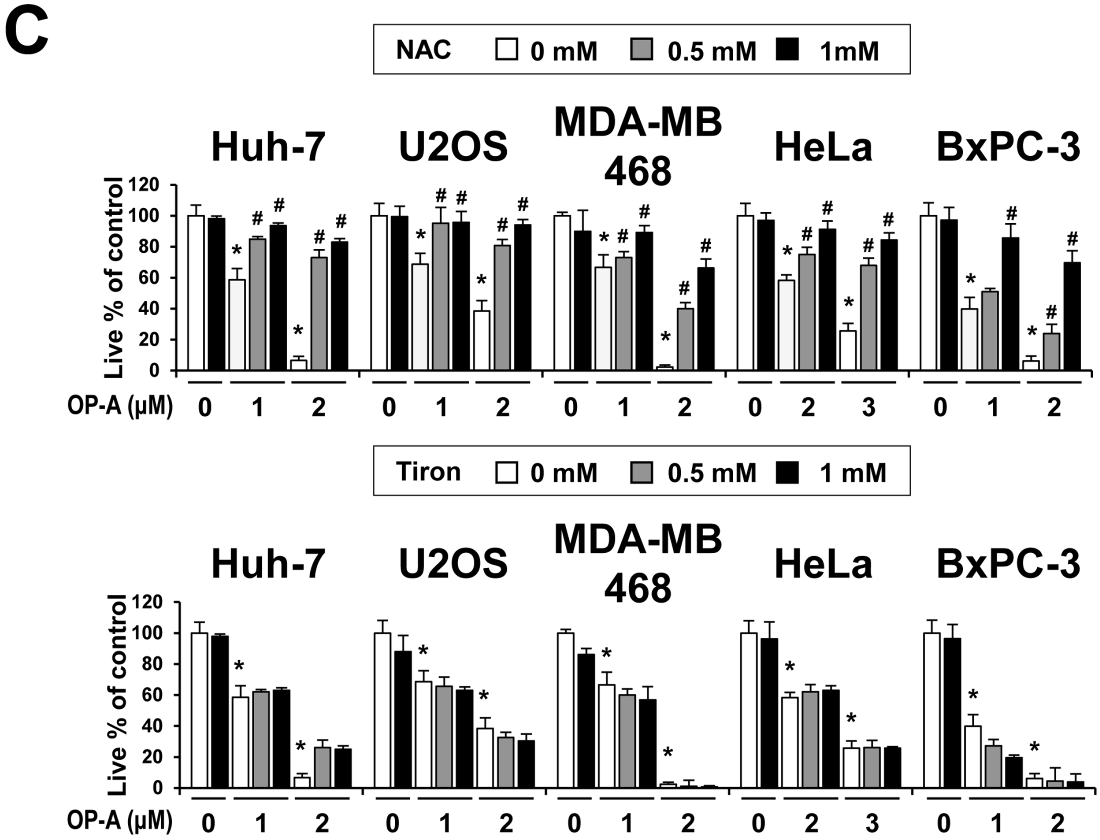

Supplementary Figure 8: OP-A-induced disruption of thiol homeostasis is critical for OP-A-induced cytotoxicity regardless of final cell death mode(s). (**A**) Cells were treated with the indicated concentrations of OP-A for 24 h. Cellular viability was assessed using calcein-AM and EthD-1. Data represent the means ± SD (n = 7). One-way ANOVA and Bonferroni's *post hoc* test. $^{*}P < 0.01$, $^{**}P < 0.001$ vs. untreated control. IC_{50}s were calculated using GraphPad Prism. (**B**, **C**) Cells were pretreated with z-VAD-fmk, necrostatin-1, CHX (B), NAC, or Tiron (C) for 30 min and further treated with OP-A at the indicated concentrations for 24 h. Cell viability was assessed using calcein-AM and EthD-1. Data represent the means ± SD (n = 7). One-way ANOVA and Bonferroni's *post hoc* test. $^{*}P < 0.001$ vs. untreated control, $^{\#}P < 0.05$ vs. OP-A treatment.

A

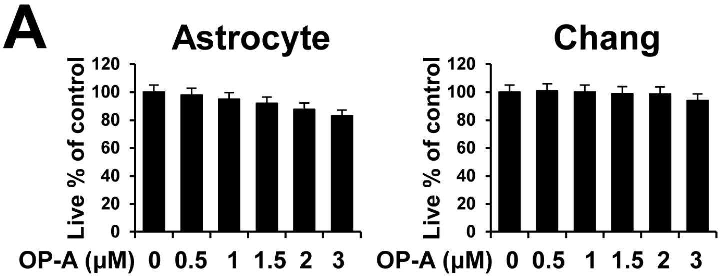

Normal cell lines	IC_{50} (μM)
**Astrocyte**	8.546
**Chang**	25.369

B

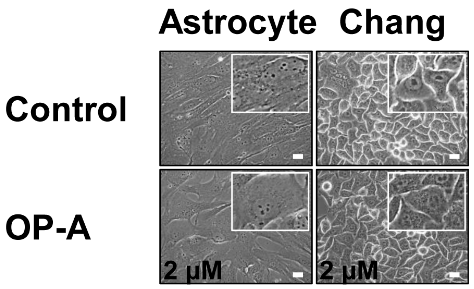

Supplementary Figure 9: Effect of OP-A on the viabilities of astrocytes and Chang liver cells. Astrocytes were obtained from Thermo Fisher Scientific (Waltham, MA, USA) and Chang normal liver cells were purchased from Sigma Aldrich. (**A**) After culturing of the cells in DMEM supplemented with 10% FBS, cells were treated with the indicated concentrations of OP-A for 24 h. Cellular viability was assessed using calcein-AM and EthD-1. Data represent the means ± SD (n = 7). Bonferroni's tests following One-way ANOVA did not reveal any significant post-hoc effects. IC^{50}s were calculated using GraphPad Prism. (**B**) Cells were observed under the phase-contrast microscope. Bar 20 μm.
